# How to use an extensive Flammer syndrome phenotyping for a holistic protection against health-to-disease transition — facts and practical recommendations

**DOI:** 10.1007/s13167-025-00423-6

**Published:** 2025-09-04

**Authors:** Olga Golubnitschaja

**Affiliations:** Predictive, Preventive and Personalised (3P) Medicine, University Hospital Bonn, Rheinische Friedrich-Wilhelms-Universität Bonn, 53127 Bonn, Germany

**Keywords:** Flammer syndrome phenotype (FSP), Sympathoexcitation, Parasympathetic-sympathetic imbalance, Sympathetic overdrive, Ischemia-reperfusion, Endothelin-1, Homocysteine, Small vessel disease, Paradigm shift, Predictive preventive personalised medicine (PPPM / 3PM), Mitochondrial stress and burnout, Health risk assessment, AI, Health-to-disease transition, Holistic approach, Suboptimal health, Phenotyping, Systemic effects, Tear fluid, Mitophagy, Individualised protection, Primary care, Population screening, Health policy

## Abstract

Due to their phenotype-associated attitude predominantly oriented towards high performance, Flammer syndrome (FS) phenotype carriers are blessed to a successful career in corresponding professional branches. This advantage is however associated with significant health risks. FSP carriers are extremely stress-sensitive. Corresponding pathways are epigenetically regulated, and modifiable risk factors are associated with the phenotype-specific psycho-somatic patterns such as a drive for meticulousness, perfectionism and exercised rigour applying strictness, discipline, or thoroughness to their own behaviour and actions. The FS phenotype is typically characterised by chronication of the transient sympathoexcitation and its dominance over parasympathetic relaxation. Chronification of the parasympathetic-sympathetic imbalance in form of sympathetic overdrive leads to chronic ischemic events in peripheral vessels and progressing tissue damage associated with the cyclic ischemia-reperfusion. Ischemic damage can be roughly estimated by levels of the vasoconstrictor endotelin-1 (ET-1) measured in blood. However, other risk factors on the one hand and compensatory mechanisms on the other hand are decisive for the damage extent at individual level. For example, chronically increased ET-1 and even mild hyperhomocysteinaemia synergistically may cause a progressing disease of small vessels, systemic inflammation and chronification of mitochondrial stress potentially resulting in chronic fatigue and mitochondrial burnout with a spectrum of associated pathologies in affected individuals. That is why predictive diagnostics utilising comprehensive individualised patient profiles are crucial for the cost-effective targeted prevention and creation of personalised treatment algorithms. Due to the high level of algorithms’ complexity, an application of AI is essential. FS is usually established early in life during pubertal maturation of otherwise healthy individuals. Therefore, FS phenotyping is instrumental for 3PM-guided cost-effective primary healthcare. To meet the needs of this patient cohort, an application of the digital health monitoring including records of mitochondrial homeostasis is strongly recommended to protect the FS phenotype carriers against health-to-disease transition. To this end, patient friendly non-invasive approach is already established utilising tear fluid multi-omics, mitochondria as vital biosensors and AI-based multi-professional data interpretation; the approach is offered to the FS phenotype carriers.

## A short history of establishing FS in the context of glaucoma research

Flammer syndrome is named by the Swiss ophthalmologist, Prof. Dr. Josef Flammer who observed and described a characteristic pattern of symptoms and signs appearing more frequently in the cohort of vasospastic normal-tension glaucoma (VNTG) patients compared to the general population. An international group of scientists in their EPMA J. 2014 publication, for the first time reached a consensus to the FS phenotype (FSP) presentation as being of significant clinical utility [1]. In 2017, Josef Flammer was awarded by the European Association for Predictive, Preventive and Personalised Medicine, EPMA, Brussels for his discovery: EPMA AWARD 2017 of excellence in research and particular achievements in medical sciences benefiting the patient and healthcare.

My FS-associated research began in the late 1990s demonstrating systemic effects detected at molecular level by gene-hunting technologies (subtractive hybridisation) in circulating leukocytes of patients, who that time were broadly referred to as vasospastic individuals (VI) with and without normal-tension glaucoma [2]. This was a fundamental step forwards to establishing FS as a group of VI with characteristic molecular profiles of systemic nature and at high risk of glaucoma. The characteristic molecular signature of VNTG patients demonstrated predisposition to extensively shifted hormonal and epigenetic regulation, methylation status of CpG islands, mRNA editing, post-translational modification, energy metabolism, stress and immune response, phosphorylation status, insufficient DNA repair capacity, excessive apoptosis and tissue remodelling, activation of proteasome activity and neurodegenerative pathways as well as altered multi-drug resistance protein core and blood-brain-barrier permeability, amongst others [2, 3]. This broad spectrum of molecular pathways involved led to the assumption that VNTG is not the only pathology, which FS may predispose affected individuals to.

## FS phenotyping as the tool for paradigm change from reactive approach to proactive healthcare

By performing a series of research projects dedicated to FSP-associated health risks, it was getting more and more evident that FSP is findable in both

A Non-diseased VI in suboptimal health conditions (pronounced stress-provoked vasoconstriction, dry mouth syndrome, vaginal dryness, high stress sensitivity, shifted regulation of sense-receptors, shifted circadian rhythms and sleep patterns, high endothelin-1 level in blood, frequent tiredness and dizziness, anxiety and panic attacks, amongst others) [1, 4–9], and
B VI with a spectrum of pathologies including pregnancy-associated risks, migraine, eye migraine, tinnitus, NTG, lacunar ischemic lesions and ischemic stroke at young age, “long COVID”, chronic inflammation, impaired wound healing, aggressive malignancies with metastatic disease etc. [10–19].

This evidence-based apparentness opened the door for potential utility of the FS phenotyping to protecting FSP carriers against health-to-disease transition at the level of reversible damage to health. For doing this, an extent of the phenotype-specific symptoms reflected e.g. in the FSP scoring, rather than currently applied descriptive approach, is instrumental for the paradigm change from reactive to 3P medicine (predictive diagnostics, targeted prevention and treatments tailored to individualised patient profiling).

## Mitochondria as a vital biosensor

In the above-presented context, what might be the feasible and reliable target for diagnostics and treatments tailored to an individualised patient profile? The field-dedicated 3PM working group has demonstrated a reciprocity between FSP at high risk of pathologies on the one hand and, on the other hand, compromised mitochondrial health [18, 20–24]. Mitochondrial stress per evidence is characteristic for chronic ischemia-reperfusion which is one of the key pathomechanisms linked to the FSP with complications. Chronic mitochondrial stress leads to compromised mitochondrial homeostasis and diminished energy supply with systemic effects measurable at cellular, tissue and organismal levels making mitochondrial homeostasis to an attractive target for an effective health risk assessment and targeted prevention in primary (protection against health-to-disease transition) and secondary (protection against pathology progression) care [20, 25, 26]. For a practical application of the established approach, FSP cases are considered by a series of recently published articles dedicated to the mitochondrial stress and homeostasis as the target for predictive diagnostics, prevention of relevant disorders and individualised rehabilitation programmes [20, 21, 23, 27, 28].

## Conclusions and expert recommendations

Health risk assessment for the FSP carriers is frequently performed by 3PMedicon GmbH [29]. This is to summarise the accumulated experience and relevant data, namely:

- FSP carriers usually belong to a socially prosperous environment with high career expectations beginning from childhood/youth, indeed well above average completion of planned career stages;
- many hours of daily professional workload are typical for them; professional success (including but not restricted to scientists, analysts, designers, art workers, perfume makers, etc.) to a great extent is associated with hyper-activation of senses, subtle perception of circumstances and hyperbolised reaction towards stressors (fluctuating temperature, sounds, smells, colours, messages, news etc.);
- they are pedantic in carefully analysing their health issues, well informed about potential genetic predispositions (cases of cardiovascular, neuro/degenerative and oncological diseases) which engage their mind to a large extent;
- normal or even low blood pressure and a slim stature are frequent for them;
- their biological age falls significantly behind chronological one;
- systemically measured mitophagy is frequently progressing into extensive autophagy followed by mitochondrial burnout.

FSP carriers are at increased risks of:

- chronification of pain, accumulation of lacunar ischemic brain lesions and predisposition to ischemic stroke, heart micro-infarction, VNTG (especially in case of genetic predisposition in the family), mild forms of cardiac arrhythmias of unclear aetiology, minor mitral valve prolapses, stress-triggered sporadic arterial stiffness and predisposition to aneurysms linked to co-diagnosed connective tissue deficits;
- often there is a delayed process of wound healing accompanied with systemic pro-inflammatory processes such as chronic inflammation and cytokine over-excitation observed e.g. in patients suffering from “long COVID”;
- in case of significant genetic predisposition in the family to malignant transformation FSP carriers are predisposed to aggressive cancer subtypes;
- frequent cases of complicated pregnancy – pre-pregnancy check-up is strongly recommended;
- in general, due to increased stress sensitivity of the FSP carriers, physiotherapeutic and well-being procedures should conform to the FSP-adapted recommendations; for example, due to their strongly pronounced vasospastic reaction towards cold stress provocation, physiotherapeutic measures utilising contrast shower and cold water/temperatures are not recommended for the FSP carriers;
- due to shifted multi-drug resistance, drugs application should be individually adapted in case of FSP.

In summary, due to their phenotype-associated attitude predominantly oriented towards high performance, FSP carriers are blessed to a successful career in corresponding professional branches. This advantage is however associated with significant health risks. FSP carriers are extremely stress-sensitive. Corresponding pathways are epigenetically regulated, and modifiable risk factors are associated with the phenotype-specific psycho-somatic patterns such as a drive for meticulousness, perfectionism and exercised rigour applying strictness, discipline, or thoroughness to their own behaviour and actions. The phenotype is typically characterised by chronication of the transient sympathoexcitation and its dominance over parasympathetic relaxation. Chronification of the parasympathetic-sympathetic imbalance in form of sympathetic overdrive leads to chronic ischemic events in peripheral vessels and progressing tissue damage associated with the cyclic ischemia-reperfusion. Ischemic damage can be roughly estimated by levels of the vasoconstrictor endotelin-1 (ET-1) measured in blood. However, other risk factors on the one hand and compensatory mechanisms on the other hand are decisive for the damage extent at individual level. For example, chronically increased ET-1 and even mild hyperhomocysteinaemia synergistically may cause a progressing disease of small vessels, systemic (low-grade) inflammation and chronification of mitochondrial stress potentially resulting in chronic fatigue and mitochondrial burnout with a spectrum of associated pathologies in affected individuals [30, 31]. That is why predictive diagnostics utilising comprehensive individualised patient profiles are crucial for the cost-effective targeted prevention and creation of personalised treatment algorithms. Due to the high level of algorithms’ complexity, an application of AI is essential [20].

**Fig. 1:**
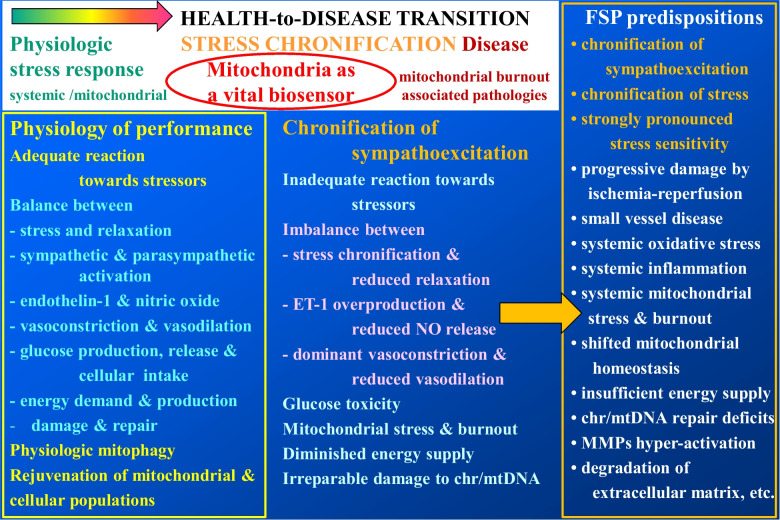
Health-to-disease transition by stress chronification; FSP is predisposed to chronification of stress and parasympathetic-sympathetic imbalance in form of sympathetic overdrive with downstream developing pathologies; pathomechanisms are detailed in the figure; therefore, the FS phenotyping is instrumental for paradigm shift from reactive medical approach to comprehensive health risk assessment, and targeted protection against disease manifestation and progression; the stage of suboptimal health is considered a reversible damage for cost-effective treatments tailored to individualised patient profiles; patient friendly non-invasive approach utilising tear fluid multi-omics and mitochondria as vital biosensors is established [20, 29]

FS is usually established early in life during pubertal maturation of otherwise healthy individuals. Therefore, FS phenotyping is instrumental for 3PM-guided cost-effective primary healthcare [32]. To meet the needs of this patient cohort, an application of the digital health monitoring including records of mitochondrial homeostasis are strongly recommended to protect the FS phenotype carriers against health-to-disease transition. To this end, patient friendly non-invasive approach is already established utilising tear fluid multi-omics, mitochondria as vital biosensors and AI-based multi-professional data interpretation; the approach is offered to the FS phenotype carriers [20, 29]. Figure 1 summarises incorporation of the FS phenotyping in paradigm shift from reactive medical services to the cost-effective 3PM meeting needs of this patient cohort:

## Data Availability

All the data used in this study are presented in this article.

## Abbreviations

AI: Artificial Intelligence
ET-1: Endothelin 1
FS: Flammer Syndrome
FSP: Flammer Snydrome Phenoype
VI: Vasospastic Individuals
VNTG: Vasospastic Normal-Tension Glaucoma
